# Complete mitochondrial genome of the jellyfish, *Chrysaora pacifica* (Goette, 1886) (Cnidaria, Scyphozoa) and the phylogenetic relationship in the related species

**DOI:** 10.1080/23802359.2019.1704648

**Published:** 2020-01-08

**Authors:** Yantao Wang, Jiehui Yin

**Affiliations:** a Key Laboratory of Marine Ecology and Environmental Sciences, Institute of Oceanology, Center for Ocean Mega-Science, Chinese Academy of Sciences, Qingdao, P. R. China;; b Laboratory for Marine Ecology and Environmental Science, Qingdao National Laboratory for Marine Science and Technology, Qingdao, P. R. China;; c Ocean College Yan Tai University, Yan Tai, China;

**Keywords:** *Chrysaora pacifica*, complete mitochondrial genome, phylogenetic relationship

## Abstract

The complete mitochondrial genome sequences of giant jellyfish *Chrysaora pacifica*, a scyphozoan species inhabiting the Bohai Sea water in China, is firstly described and analyzed in this research. The mitogenome is a circular molecule 16,964 bp in length, including 13 protein-coding genes (Cox 1, Cox2, Atp 8, Atp 6, Cox 3, ND2, ND5, ND 6, ND3, ND4L,ND1,ND4, Cob), 2 tRNAs (trnW, trnM), 2 rRNA genes (small subunit RNA and large subunit RNA). The neighbor-joining (NJ) phylogenetic tree in the related species showed that *C. pacifica* is close to *Chrysaora quinquecirrha*.

*Chrysaora pacifica* (Goette,1886) (Cnidaria, Scyphozoa) is distributed in North Pacific Ocean, and most coastal waters in Korea and usually appeared from May to September (Lee et al. 2016). It was seen in the Bohai Sea water in China since the past two years.

It is imperative to analyze the complete mitochondrial genome of scyphozoan species to better understand the molecular phylogenetic relationship between Cnidaria species. However, there were only several species involved until now, such as *Aurelia* spp. (Hwang et al. 2014; Shao et al. 2006), *Craspedacusta sowerbyi* (Zou et al. 2012), *Chrysaora quinquecirrha* (Hwang et al. 2014), *Nemopilema nomurai*, and *Rhopilema esculentum* (Wang and Sun 2017a, 2017b) *Parumbrosa polylobata* (Feng et al. 2019). In this study, we firstly report the complete mitochondrial genome from *C. pacifica* to obtain the basic genetic information of *C. pacifica* population in Bohai Sea, China. It is expected that the information obtained from complete mitochondrial genome sequence of *C. pacifica* would provide a useful genetic resource to be utilized in the future investigation on population genetics and phylogenomics of Scyphozoa. DNA from about 1 g bell tissue of a single specimen of *C. pacifica* with bell diameter of 10 cm collected from Bohai Sea, China, (40.14 N, 121.91E) was extracted by the standard phenol-chloroform extraction method (Sambrook and Russell 2001) and part of the rest of the sample was preserved in 20°C at KLMEES Institute of Oceanology with the voucher no. Cmit001.

The complete mitochondrial genome of *C. pacifica* was 16,964 bp in length and the GenBank accession No. is MN448506. It consists of 13 protein-coding genes (including Cox 1, Cox2, Atp 8, Atp 6, Cox 3, ND2, ND5, ND 6, ND3, ND4L, ND1, ND4, Cob), 2 tRNAs (trnW, trnM), 2 rRNA genes (small subunit RNA and large subunit RNA). rRNA genes (small subunit RNA and large subunit RNA). All the genes showed complete stop codons using TAA and TAG. There is also a slight anti-G bias (9.23%) on the 3rd position of all the genes. The start codon of ND3 and ND6 are different from other species, such as *A. aurita* (Shao et al. 2006).

The mitochondrial genome base composition for 13 genes was 30.97% for A, 38.05% for T, 14.99% for G and 15.99% for C. The A + T base composition (69.02%) was higher than G + C (30.98%) based on the sequences of 13 genes, suggesting that the *C. pacifica* has low G + C ratio in the mitochondrial genome. The neighbor-joining (NJ) phylogenetic tree among 9 species was generated based on the complete mitochondrial genome from NCBI (Figure 1). The results showed that that the *C. pacifica* is close to *Chrysaora quinquecirrha* (GenBank No. NC020459.1) clustered in a separate branch, and was far related to other species.

**Figure 1.**
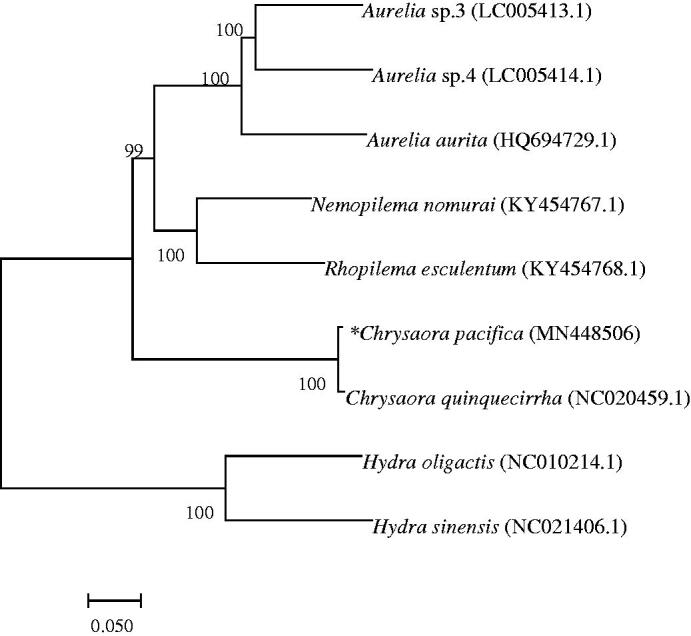
Phylogenetic relationship revealed by NJ tree.
